# Emerging Roles of NPQ/Spexin in Physiology and Pathology

**DOI:** 10.3389/fphar.2019.00457

**Published:** 2019-05-07

**Authors:** Shuang-Yu Lv, Yu-Chen Zhou, Xiao-Mei Zhang, Wei-Dong Chen, Yan-Dong Wang

**Affiliations:** ^1^ Key Laboratory of Receptors-Mediated Gene Regulation and Drug Discovery, School of Medicine, Henan University, Kaifeng, China; ^2^ Key Laboratory of Molecular Pathology, School of Basic Medical Science, Inner Mongolia Medical University, Hohhot, China; ^3^ State Key Laboratory of Chemical Resource Engineering, College of Life Science and Technology, Beijing University of Chemical Technology, Beijing, China

**Keywords:** spexin, NPQ, obesity, metabolism, neuropeptide

## Abstract

Spexin (SPX), also called neuropeptide Q (NPQ), is a novel endogenous neuropeptide. Spexin gene and protein are widely expressed in central nervous system and peripheral tissues in humans, rodents, goldfish, etc. A few of physiological and pathological roles of spexin are gradually emerged recently. This article summarized the roles of spexin in feeding behavior, gastrointestinal motility, obesity, diabetes, energy metabolism, endocrine, mental diseases, and cardiovascular function. Given the broad roles of spexin, this neuropeptide has attracted much interest from investigators and will be as a promising future target for novel therapeutic research and drug design.

## Introduction

**Figure 1:**
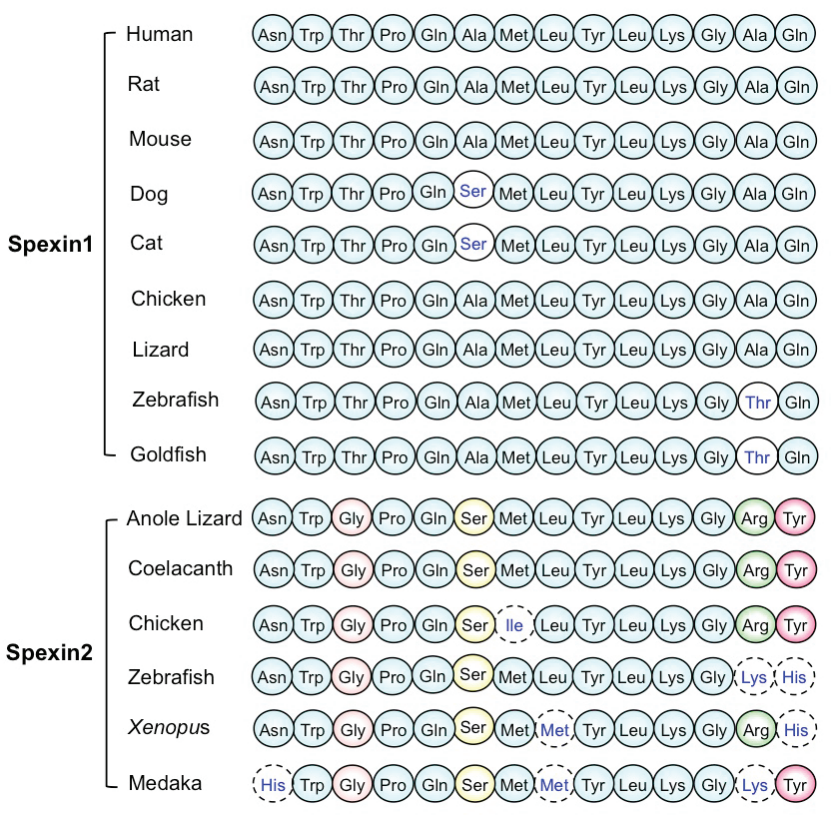
The amino acid sequence of mature spexin1 and spexin2 in various species. (The figure modified from Wong et al., 2013; Kim et al., 2014).

Spexin (SPX), namely neuropeptide Q (NPQ), is a new identified peptide hormone. Spexin was first identified in human genome through bioinformatic method based on a hidden Markov model (Mirabeau et al., 2007). Then spexin was confirmed by biochemical method and was first detected in the esophagus and stomach of mice (Mirabeau et al., 2007). The prepropeptide human spexin containing 116 amino acid residues was encoded by *C12ORF39* gene (Wan et al., 2010). In humans, the precursor of spexin contains a signal peptide, two prohormone cleavage sites, and predicted processed peptide (Sonmez et al., 2009). The small amino acid sequence between dibasic cleavage sites with C-terminal amidation was called spexin (Sonmez et al., 2009). The nuclear magnetic resonance (NMR) analysis indicates that the molecular surface of spexin (gold fish) is largely hydrophobic except for Lys^11^, and its 3D structure is an α-helix from Gln^5^ to Gln^14^ with a flexible NH_2_ terminus from Asn^1^ to Pro^4^ (Wong et al., 2013). The amino acid sequence of the spexin is highly conserved among various species (Figure 1; Wong et al., 2013), suggesting that the peptide has an essential property during biological evolution and may be potentially involved in regulating a variety of physiological and pathological functions. The spexin paralogous gene (*spexin2*) was identified in vertebrate chromosomes. However, mammals do not have *spexin2*, like *spexin* (namely *spexin1*). *Spexin2* encodes a mature peptide containing 14 amino acids with amidation at the C terminus, named spexin2 (Figure 1; Kim et al., 2014).

Spexin was proved to be a natural ligand for galanin receptor 2/3 (GALR2/3) (Kim et al., 2014). The receptor-ligand binding assay demonstrates that it activated humans, *Xenopus*, and zebrafish GALR2/3 family receptors but not GALR1 (Kim et al., 2014). Reyes-Alcaraz et al. found that the spexin and galanin induce specific active conformations of GALR2, indicating that the two ligands activate differential signaling *via* the same receptor, GALR2 (Reyes-Alcaraz et al., 2018). Galanin, as a classic neuropeptide, has a regulation on numerous physiologic and pathophysiological processes, including feeding and energy homeostasis, osmotic regulation and water intake, pain, regeneration and neurite outgrowth, Alzheimer’s disease, cerebral ischemia and stroke, seizures and epilepsy, anxiety disorders, depression, etc. (Lang et al., 2015).

Spexin mRNA and protein are widely distributed in central nervous system (CNS) and peripheral tissues in various species. In humans, spexin mRNA and spexin cytoplasmic immunoreactivity were found in skin, lung, stomach, small intestine, colon, liver, pancreatic islets, thyroid, adrenal gland, visceral fat, kidney, etc. (Gu et al., 2015). In rats, high levels of mRNA were detected in brain, hypothalamus, esophagus, liver, kidney, thyroid, and ovary (Porzionato et al., 2010). The rat spexin-like immunoreactivity was strongly determined in epidermis and sebaceous glands of skin, epithelium of esophagus, base of glands in stomach, epithelium of small intestine, hepatocytes, etc. (Porzionato et al., 2010). In goldfish, spexin mRNA was confirmed in CNS and peripheral organs, especially in the optic tectum, hypothalamus, brain stem, liver, and testis (Wong et al., 2013). The broad distribution of spexin suggests multiple physiological and pathological functions of spexin.

## Feeding and Energy Metabolism

In Ya-fish, the *spexin* mRNA level in forebrain of postprandial group was higher than preprandial group (Wu et al., 2015). The mRNA expression of spexin in forebrain was reduced in the fasting group comparing with the feeding group, while the spexin mRNA was elevated after refeeding at the 7th day comparing with the feeding group. The spexin mRNA in forebrain was determined by different feeding status in Ya-fish, suggesting that spexin gene expression was controlled by different feeding conditions or metabolic status (Wu et al., 2015). Deng et al. have cloned and analyzed *spexin* gene from spotted scat (*Scatophagus argus*). And it was reported that food deprivation upregulated the *spexin* mRNA level in hypothalamus of spotted scat; however, the *spexin* mRNA level was decreased after refeeding for the unfeeding group, compared with the still unfeeding (fasting) group (Deng et al., 2018). In flatfish, the *spexin* mRNA in the hypothalamus was upregulated under the fasted condition (Wang et al., 2018). Wu et al. found that the contents of insulin and spexin in plasma and the mRNA levels of insulin and spexin in liver were all increased after refeeding in the 3-day-fasted goldfish, comparing with unfeeding group (Ma et al., 2017). Intraperitoneal (i.p.) and intracerebroventricular (i.c.v.) injection of spexin reduced food intake during a 2-h feeding period in goldfish (Wong et al., 2013). And i.c.v. spexin inhibited the increase of food intake induced by neuropeptide (NPY) and orexin treatment. The inhibitory effect of central spexin on goldfish feeding behavior was induced by downregulation of orexigenic factors, including NPY, agouti gene-related protein (AgRP) and apelin, and upregulation of anorexigenic factors, including cholecystokinin (CCK), proopiomelanocortin (POMC), amphetamine-regulated transcript (CART), melanin-concentrating hormone (MCH), and corticotropin-releasing hormone (CRH; Wong et al., 2013). In orange-spotted grouper, *spexin* mRNA in hypothalamus was upregulated by 7 days of food deprivation, whereas the *spexin* gene was downregulated after refeeding treatment, compared with the group fasted for 7 days (Li et al., 2016). Peripheral injection of spexin stimulated *pomc* mRNA expression and inhibited *orexin* mRNA expression in hypothalamus of the orange-spotted grouper (Li et al., 2016). Zheng et al. found that *spx1* knockout Zebrafish exhibited a higher food intake than the wild type (WT; Zheng et al., 2017). The agouti-related protein 1 (*agrp1*) gene expression was higher in *spx1* ^−/−^ mutant fish than in WT fish after feeding, and intracranial treatment with spexin1 decreased the *agrp1* gene expression in the zebrafish hypothalamus (Zheng et al., 2017). This result indicates that the inhibitory effect of spexin1 on food intake might be medicated by *agrp1* gene (Zheng et al., 2017). In fish, under the fasting condition, the *spexin* gene was decreased in the forebrain of Ya-fish, whereas it was increased in the hypothalamus of spotted scat, flatfish, and orange-spotted grouper, comparing with the fed control. And the increased/decreased *spexin* gene expression of the fasting group was recovered when the food was available again (refeeding). Central or peripheral administration of spexin reduced food intake in goldfish.

In diet-induced obesity (DIO) rats, chronic subcutaneous (s.c.) injection with spexin reduced food intake (Walewski et al., 2014). These results indicate a solid inhibitory effect of exogenous spexin on food intake as well as a closed relationship between spexin and food control.

It is well established that the long-chain fatty acid (LCFA) uptake and storage are a key factor for body weight control (Petrescu et al., 2005). Spexin treatment produced a suppression effect on LCFA uptake into adipocytes isolated from untreated DIO mice (Walewski et al., 2014). Chronic i.p. injection of spexin reduced the respiratory exchange ratio (RER) at night and increased locomotor activity in DIO mice (Walewski et al., 2014). These results could explain the inhibitory effect of spexin on the body weight of mice with obesity. Recent report shows that chronic i.p. injection with spexin diminished hepatic lipids, serum alanine aminotransferase (ALT), and aspartate aminotransferase (AST) in mice with hepatic steatosis/nonalcoholic fatty liver disease (HS/NAFLD; Jasmine et al., 2016). In hepatocytes isolated from DIO mice, spexin treatment reduced LCFA uptake (Jasmine et al., 2016). These results demonstrate a potentially beneficial effect of spexin in NAFLD treatment. *In vitro*, the high level of insulin induced by glucose stimulated *spexin* gene expression in both goldfish hepatocytes and brain cells (Ma et al., 2017). Human study shows that the serum spexin was negatively correlated with age, BMI, fasting glucose, and TG in healthy adult women, suggesting that spexin could independently predict the risk of high BMI and high fasting glucose (Lin et al., 2018a). In human adipocytes and murine 3 T3-L1 cells, spexin exhibited a stimulatory effect on lipolysis and an inhibitory effect on lipogenesis and glucose uptake (Kolodziejski et al., 2018). Clinical study shows that the serum spexin had a modest association with components of metabolic syndrome (MetS) only in women (Al-Daghri et al., 2018b). The suppression of spexin on lipogenesis, LCFA uptake, and RER and the promotion of spexin on lipolysis and motor activity indicate that spexin is a key negative regulator for energy budget.

## Obesity and Diabetes

Spexin is a potentially regulating factor in obesity and energy metabolism (Wang et al., 2016). After daily injection (i.p.) with spexin for 6 days, the body weight of the high-fat-diet mice was reduced (Walewski and Berk, 2013). Clinical study shows that the spexin gene and protein were downregulated in omental and subcutaneous fat in obese patients compared with the normal fat (Walewski and Berk, 2013; Walewski et al., 2014). The concentration of serum spexin in obese patients was lower than that of the healthy participants (Walewski and Berk, 2013). Moreover, the serum spexin was markedly negative correlation with leptin in the obese patients (Walewski and Berk, 2013; Walewski et al., 2014). In children, the circulating serum spexin level in obese people was lower than that of the normal weight person (Kumar et al., 2016; Chen et al., 2019), indicating a potential role of spexin in childhood obesity. The circulating spexin level was inversely correlated with leptin in adolescents with obesity (Kumar et al., 2017). The increasing energy expenditure of spexin was in accordance with the decreasing body weight of spexin. However, the underlying mechanism needs to be further explored. More research is wanted to develop a spexin-based antiobesity drug.

In human tissues, the spexin immunoreactive cells and *spexin* mRNA were detected in endocrine and epithelial tissues, such as adrenal gland, visceral fat, thyroid, liver, etc. (Gu et al., 2015). The results indicate a potential role of spexin in modulating metabolism. Karaca et al. found that patients with type 1 diabetes mellitus (T1DM) have a lower level of spexin, not related to body mass index (BMI), glucose, or lipid parameters, comparing with healthy subjects (Karaca et al., 2018). Serum spexin levels in type 2 diabetes mellitus (T2DM) were lower than that of healthy subjects, and spexin was negatively correlated with blood glucose, triglyceride (TG), hemoglobin A1c (HbA1c), and low-density lipoprotein-cholesterol (LDL-C; Gu et al., 2015, 2016). However, Hodges et al. found that the serum spexin concentration did not show difference between the three groups (normal weight, obese, and obese with T2DM) in adolescents (Hodges et al., 2018). Recently, it was shown that the level of spexin exhibited a notably increase after 6 months in the pregnant women with gestational diabetes mellitus (GDM; Al-Daghri et al., 2019).

In adolescent patients with obesity or T2MD, the circulating spexin level is inversely correlated with blood glucose, leptin, lipids, etc. However, in adult patients with obesity or T2MD, the circulating spexin level does not exhibit a correlation with blood glucose, leptin, triglyceride (TG), etc. (Hodges et al., 2018). These results indicate that spexin exerts different pathological effects on obesity and metabolism between adults and adolescents. Al-Daghri et al. show that the level of spexin in serum had a modestly relationship with glucose and insulin sensitivity in pregnant women but had no influence on GDM and obesity (Al-Daghri et al., 2018a). In obese children, spexin was negatively correlated with insulin resistance and pancreatic β-cell function indicators (Chen et al., 2019). Recently, Sassek et al. have shown that spexin not only inhibited the insulin secretion from cultured cells and isolated islets induced by glucose *in vitro* but also reduced the insulin secretion in obese rats *in vivo* (Sassek et al., 2018a). Sassek et al. found that spexin was located inside the β cells of the pig pancreas, and the release of spexin from islets was increased after a short term and decreased after a long term following treatment with glucose (Sassek et al., 2018b). In addition, spexin could improve the cell viability and proliferation of pancreatic islet cells and upregulate the protein level of proliferating cell nuclear antigen (PCNA; Sassek et al., 2018a).

## Gastrointestinal Motility and Bile Acid

The *spexin* mRNA was located in the submucosal layer of esophagus and stomach fundus of mouse using hybridization *in situ* (Mirabeau et al., 2007). The rat stomach explant assay indicates that spexin caused a contractile activity of stomach muscle (Mirabeau et al., 2007). The result indicates a potential role of spexin in regulation of digestive tract function. Spexin (i.p.) increased the mouse intestinal transit rate and promoted the colonic bead expulsion *in vivo*, and spexin obviously stimulates the intestinal/colonic muscular contractions *in vitro* (Lin et al., 2015). Spexin exerts the stimulatory effect on bowel motility through GALR2 receptor by activating L-type voltage-dependent calcium channel (Lin et al., 2015). The findings demonstrate that spexin is an important regulator in gastrointestinal function. However, whether the central spexin has a similar effect on gastrointestinal motility and whether the effect of spexin on bowel motility via CNS needs to be identified in the future study.

Current study shows that spexin was involved in bile acid synthesis (Lin et al., 2018b). Peripheral (i.p.) treatment with spexin decreased the total bile acid level and cholesterol 7α-hydroxylase 1 (*CYP7A1*) mRNA level in the mouse liver, and this effect could be blocked by the GALR2 and GALR3 antagonists (Lin et al., 2018b).

## Endocrine Regulation

Recent report shows that spexin may be a neuroendocrine signal related to multiple functions (Ma et al., 2018). In zebrafish, *spexin* mRNA was highly expressed in brain and ovary. Incubated with spexin obviously inhibits release of luteinizing hormone (LH) in cultured goldfish pituitary cells, and i.p. injection with spexin reduced goldfish serum LH levels (Liu et al., 2013). The *spexin* gene expression in hypothalamus was affected by gonadal hormones in female goldfishes (Liu et al., 2013), implicating a potential role of spexin in reproduction and endocrine. Trudeau proposed that spexin, as an inhibitory neuropeptide, regulates LH in teleosts (Trudeau, 2018). Wang et al. have cloned the *spexin* gene from a flatfish, the half-smooth tongue sole (*Cynoglossus semilaevis*; Wang et al., 2018). Treatment (i.p) with spexin upregulated the *gnih* (gonadotropin inhibitory hormone) and *gnrh3* (gonadotropin-releasing hormone 3) gene expression and downregulated the *gh* (growth hormone), *fshβ* (follicle-stimulating hormone β), and *gthα* (gonadotropin hormone *α*) gene expression in the hypothalamus of the half-smooth tongue sole (Wang et al., 2018). Treatment with 17β-estradiol (E2) decreased the *spexin* gene expression in the hypothalamus of the spotted scat *in vitro* and *in vivo* (Deng et al., 2018). These results suggest that spexin played a role in regulating reproductive function, especially inhibiting the LH secretion. However, all the present research has only utilized fish model. The animal models of mammalian and clinical studies are needed in the future.

An *in vitro* experiment shows that the incubation with spexin increased aldosterone secretion in rat isolated zona glomerulosa (ZG) and stimulated corticosterone secretion in rat adrenocortical primary cells (Rucinski et al., 2010), indicating that spexin was involved in controlling the adrenocortical secretory function. In addition, BrdU assay shows that spexin inhibited adrenocortical cell proliferation (Rucinski et al., 2010).

## Pain, Anxiety, and Depression

Currently, spexin was reported to play a role in pain, anxiety, and depression. I.c.v. administration of spexin produced antinociceptive effect in the mouse warm water tail withdrawal assay (Toll et al., 2012). Female rats injected with spexin in hippocampal CA1 show a decrease in pain sensitivity in the formalin test, indicating an analgesic effect on tonic pain (Pirzeh and Taherianfard, 2014). Intra hippocampal CA3 (IHCA3) administration of spexin inhibited the pain sensitivity in the both early and late phrases of the rat formalin test (Moazen et al., 2017). Lv et al. found that centrally administrated spexin produced antinociceptive effect against inflammatory pain by dynorphin/κ-opioid receptor pathway (Lv et al., 2019). All of the results indicate the analgesic effect of central spexin; however, the effect of spexin at the peripheral level is not clear. A comparison between spexin and morphine in antinociceptive efficiency needs to be evaluated. The synergistic analgesic relationship of morphine and spexin is still unknown.

Reyes-Alcaraz et al. demonstrate that i.c.v. injection with spexin-based GALR2-specific agonists induced an anxiolytic effect in mice (Reyes-Alcaraz et al., 2016). Chronic i.p. injection of SSRI antidepressant escitalopram caused a decrease in *spexin* gene expression in hypothalamus and an increase in *spexin* gene expression in the hippocampus and striatum in rats (Palasz et al., 2016). This study suggests that spexin was involved in regulating the depression. Palasz et al. found that chronic i.p. administration with haloperidol and chlorpromazine, the classical antipsychotic drugs, increased the *spexin* and *pomc* mRNA levels while decreased the *kisspeptin-1* mRNA level in the rat amygdala (Palasz et al., 2018). It indicates that the neuropeptide spexin in the amygdala may be related to the mechanism of the antipsychotic drugs.

## Other Roles

Rats injected (i.c.v.) with spexin exhibited an increase in mean arterial pressure, a decrease in heart rate, and a marked decrease in urine flow rate (Toll et al., 2012), indicating that central spexin was involved in modulating the cardiovascular and renal activities. *Spexin* mRNA levels of carotid body in hyperoxia-exposed rats were higher than normoxia-exposed group, suggesting spexin in carotid body may be a regulator in hyperoxia-induced plasticity (Porzionato et al., 2012).

## Conclusion/Perspectives

**Figure 2:**
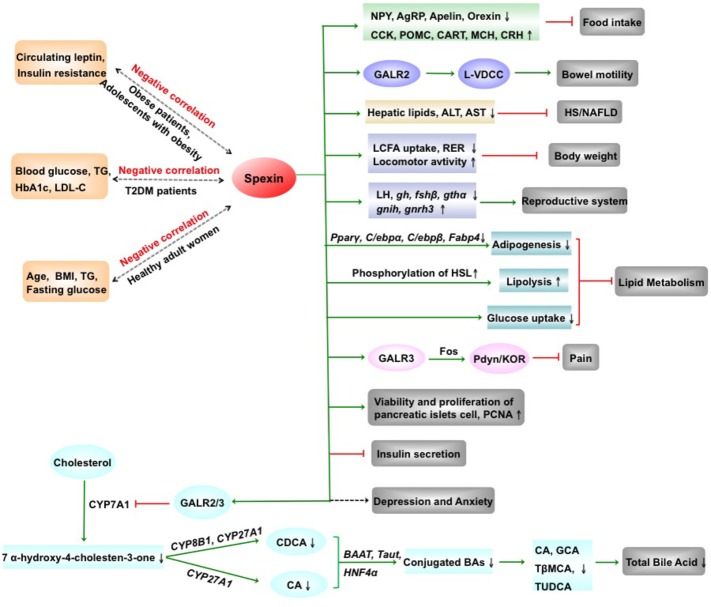
The physiological and pathophysiological effects of spexin. AgRP, agouti gene-related protein; ALT, alanine transaminase; AST, aspartate aminotransferase; BAAT, bile acid CoA: amino acid N-acyltransferase; BAs, bile acids; BMI, body mass index; CA, cholic acid; CART, cocaine-amphetamine regulated transcript; CCK, cholecystokinin; CDCA, chenodeoxycholate; CRH, corticotropin-releasing hormone; CYP27A1, sterol 27-hydroxylase; CYP7A1, cholesterol 7α-hydroxylase 1; CYP8B1, sterol 12α-hydroxylase; fshβ, follicle-stimulating hormone β; GalR2, galanin receptor 2; GalR3, galanin receptor 3; GCA, glycocholate; gh, growth hormone; gnih, gonadotropin inhibitory hormone; gnrh3, gonadotropin-releasing hormone 3; gthα, gonadotropin hormone α; HbA1c, hemoglobin A1c; HNF4α, hepatocyte nuclear factor 4 alpha; HS/NAFLD, hepatic steatosis/nonalcoholic fatty liver disease; KOR, κ-opioid receptor; LCFA, long-chain fatty acid; LDL-C, low-density lipoprotein-cholesterol; LH, luteinizing hormone; L-VDCC, L-type voltage-dependent calcium channel; MCH, melanin-concentrating hormone; NPY, neuropeptides Y; PCNA, proliferating cell nuclear antigen; Pdyn, prodynorphin; POMC, proopiomelanocortin; RER, respiratory exchange ratio; T2DM, type 2 diabetes mellitus; Tβ-MCA, tauro-β-muricholate; taut, taurine transporter gene; TG, triglyceride; TUDCA, tauroursodeoxycholate; ↑, increase; ↓, decrease; green arrow, promotion; red arrow, inhibition. (Partial figure originated from Lin et al., 2018a,b).

In summary, spexin, a novel biologically active peptide, has a broad distribution in CNS and periphery and is involved in regulating multiple physiological functions (Figure 2). Spexin inhibits food intake and body weight, promotes gastrointestinal motility, and exhibits a negative correlation with leptin in the obese patients. Serum spexin levels in T2DM are decreased and negatively correlated with blood glucose, TG, etc. The human studies are needed to be determined the potential of therapeutic strategies targeting spexin on T2DM. Spexin was shown to suppress LCFA uptake into adipocytes and alleviate hepatic injury of mice with HS/NAFLD. In addition, spexin inhibits release of LH, produces an antinociceptive effect, and is involved in modulating depression, anxiety, blood pressure, etc. Remarkably, the investigation regarding the physiological and pathological functions of spexin is still at the beginning, and the information about the molecular mechanism of the spexin is deficient. Therefore, the receptor-activated signal transduction pathways of spexin and clinical trials are needed to be explored in the future. Moreover, whether spexin could across the blood-brain barrier is still unknown. Development of the more stable and not easily degradable spexin analogues might provide us a new therapeutic tool for liver disease in the future.

## Author Contributions

S-YL, Y-CZ, and X-MZ wrote the manuscript. S-YL prepared the figures. W-DC and Y-DW edited and revised the manuscript.

### Conflict of Interest Statement

The authors declare that the research was conducted in the absence of any commercial or financial relationships that could be construed as a potential conflict of interest.
